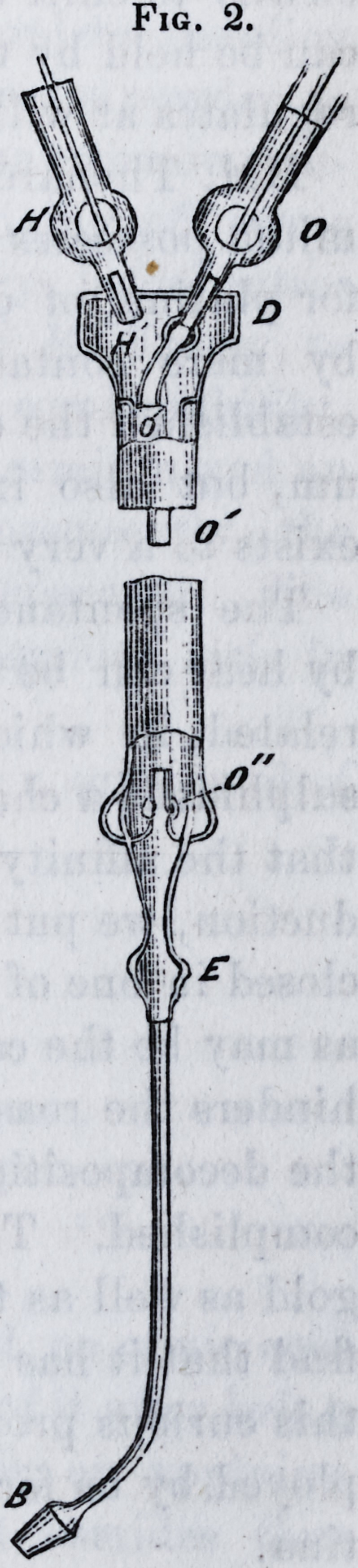# Platina and the Metals Which Accompany It

**Published:** 1860-04

**Authors:** H. Sainte Claire Deville, H. Debray


### ARTICLE V.

*Platina and the Metals which Accompany it.* By MM. H. Sainte Claire Deville and H. Debray (Translated for the American Journal of Dental Science from the *Annales de Chimie et de Physique.*)

The metallurgy of platina is an art entirely modern; the introduction of the metal into scientific and industrial laboratories being of recent date. Hence all that pertains to the metallurgy of this metal and its congeners, is in general little known, though specially deserving the attention of the chemist. His personal interest should attract him to the subject, for he is the chief consumer of platina, and to this metal he owes (with the exception of some capital experiments) the means of operating securely and promptly in the majority of chemical analyses. Up to this time, in fact, outside of chemical uses, platina has had no application of any importance. The ores of platina, when the search for them shall be better understood, or the known localities thoroughly worked, will probably not be more rare than gold; and as this metal is nearly indestructible and its high value protects it against loss and accidents of all kinds, it will ultimately accumulate in commerce, and become more and more common. Perhaps then it may be generally used where its great density and somewhat tarnished appearance will present no obstacle to its employment, and especially where its absolute inalterability will be of some importance. All these questions depend, for their solution, upon the price at which the metal can be furnished, and chemists are interested in seeing it lowered to a point which will permit the large vessels of our laboratories to be made of platina.

It is in the hope of facilitating a progress of this kind, that we have undertaken the troublesome researches, the results of which we are about to give in this memoir—

researches which have cost us more than four years of labor. If we succeed, we are sure of finding a very sweet recompense in the kind feelings of our brother-chemists whose studies we shall have facilitated, our methods permitting us to diminish hereafter the price of recovery of our precious metal. Up to the time of our first communications, no one had thought of utilizing all the metals which accompany platina in the mine, with the exception of palladium and osmium, which were always of sufficient importance to be separated, and platina has been extracted from its ores by leaving a residue which has accumulated in almost all the very few workshops of Europe, and at the Russian mint. We shall show that rhodium and iridium which enter largely into these residues, are very useful in modifying the qualities of platina. Consequently, in finding an easy and economical method for the extraction from ores of platina of all the utilizable substances in the form of an alloy of platina, rhodium and iridium, we have rendered an important service to the metallurgy of platina. We have accomplished this by a method which will be described in detail in this memoir.

To treat securely the crude ore of platina it was necessary to know its exact composition. In one of the chapters of this memoir will be found the analysis of all the known ores of platina of which we have been able to procure specimens, and at the same time a method of assay by the dry way and new processes of analysis enabling us to operate surely and expeditiously.

At the same time we have studied the principal physical properties of the alloys which are obtained by incorporating with ores of very pure platina a certain quantity of residues of platina rich in iridium or rhodium, thus making triple alloys interesting on account of their applications. To understand how to effect these mixtures, we were obliged to know—1, the composition of the residues of platina—2, the composition of the osmiurets of iridium of all the qualities of which we have been able to procure specimens. In this memoir will be found a chapter on the solution of this double question, and the dry methods for the rapid assay of residues and osmiurets, as well as methods by the wet way for the precise analysis of the osmiurets of iridium.

The general processes of the new metallurgy of platina which we propose, are exclusively the dry process and methods of fusion at a very high temperature ; they will be successively described in different chapters in which will be treated: the revivification of pure platina—the metallurgy of pure platina—the extraction from the crude ore of a triple alloy of platina, rhodium and iridium of a convenient and constant composition—of the extraction either from residues or from the osmiuret of iridium the utilizable metals which they contain—platina, palladium, iridium and rhodium.

We have succeeded (by thus operating on entirely new methods and at temperatures which have hitherto been obtained only upon comparatively small points,) in making a special study of the properties of the platinoid metals in conditions still not generally known. We shall study these properties in the first chapters which will make up the theoretical portion of this memoir ; the rest being devoted, as we have already said, to the analyses and metallurgy, will form the technical part.

We are indebted to General Samarski, chief of the mining corps of Russia, to Jacobi, the counsellor of state, to M. Kocksbarow, of the Academy of Sciences of St. Petersburg, and to Colonel de Rochette, for the majority of the specimens upon which we have operated during the course of our work. It was extremely difficult to procure the elements of our researches, which are not found in commerce, and which the mint of Russia alone possesses. These gentlemen will be pleased to accept this expression of our sincere gratitude.

We are equally indebted to MM. Desmontis and Chapuis, to M. Quenessen and to M. Pavard of Paris, and to

Mr. Mathey, of London, for a great number of specimens of residues of every kind from their shops, which have enabled us to give analytical results as complete as possible in reference to these interesting subjects.

Finally we mention with pleasure the intelligent assistance we have received during a portion of this long labor, from M. Hautefeuille, a very skillful young chemist, to whom we are happy to express our thanks.

### CHAPTER I.

### *On some Properties of the Platinoid Metals.*

The family of platinoid metals has a peculiar character which completely isolates them from other more or less natural families formed from other metals. In our opinion, it would be well to leave all the species which compose it in a single group which would admit them only, and contain them all. It is true that these metals are not all analogous in every particular, but they have a special character, a common physiognomy which will always hinder us from separating as we study them, even when under the guidance of a very rational classification.

These metals are never found separate from one another, except palladium, and that very seldom, and it is to be remarked that palladium is the one among them which approaches nearest the other metals. More or less unchangeable under the influence of oxygen and chlorine, they are similar in the facility with which they surrender to reducing agents, the elements with which they are combined. Their principal affinities are with the halogens, as bromine, chlorine, iodine and cyanogen, and they all form those compounds, characteristic of the series of platinoid metals, in which salammoniac or chloride of potassium is united with a metallic chloride to form a combination generally little soluble in an excess of ammoniacal salt, if the proportion of chlorine combined with the metal is sufficiently high.

All these bodies possess besides the curious property of determining, by their simple contact, a great number of chemical reactions; a catalytic action, as Berzelius called it, which has been used for experiments of the utmost importance to chemistry. It must not be supposed that phenomena of this kind can be wholly attributed to the porous condition of those metals which are only known in the form of sponge. Platina melted and beaten out under the hammer is as active in this way as that obtained by the aggregation of its sponge.

The differences among these bodies are equally remarkable. Thus osmium, which burns in the air to form osmic acid vapor, has been compared to arsenic by Berzelius; while recently Dumas inclines to place it side by side with tellurium. It is certainly a metalloid, the metalloid of the platina series.

Ruthenium, with which we have quite recently become acquainted through the admirable researches of M. Claus, and the oxyd of which M. Fremy has recently obtained in a crystalline form, approaches tin in its chemical properties and even in the form of this oxyd, which is a prism with a square base identical with the prism of oxyd of tin.

We shall show that palladium is analogous to silver by a great number of characters; its volatility, its oxydizability at a properly regulated temperature, which approaches it also to mercury; its action on hydriodic acid, &c.; but the basic energy of oxyd of silver is found in none of the oxyds of palladium.

Rhodium is a metal which can be compared to no other. Placed near silver on account of its oxydizability when hot, the basic properties of its principal oxyd, and the remarkable effect which sulphuric acid or rather bisulphate of potash exerts upon it; near gold, by the reactions of its chlorides, it is difficult to make it a metal less noble than gold, on account of its resistance to the action of aqua regia.

Platina, in all its properties, is the true analogue of gold, and whenever the platinoid metals have not been classed together, platina has always been ranged with gold.

The common metals present but few analogies with iridium, which is superior to them all by the resistance it offers to the majority of our most energetic reagents; and certainly if the physical properties of iridium corresponded with its chemical characters, it would be the king of the metals rather than gold.

All these considerations induce us to form of the metals of platina a group of which every species shall have its analogue among the common metals, and this family will be as natural as is, among mammalia, the family of marsupials, composed of insectivora, rodents, carnivora, &c., which their common physiognomy, in sufficiently important characters, prevents us from distributing in the series of mammalia, so well arranged by Cuvier.

Before examining successively the platinoid metals which we have studied, we must be permitted to remark how incomplete the history of the physical properties of certain simple substances, and especially of the platinoid metals has been left, so that, in reference to that which it would seem the easiest to determine, density, we find usually in chemical treatises only discordant indications. Berzelius, operating on the small quantities of material which he had at his disposal, was content with discovering the simple bodies, their important reactions, their equivalents, and after those magnificent researches which every body knows, he seemed to disdain the radical he had isolated, and generally submitted it only to the action of reagents, neglecting to study its form and the modifications it might receive from physical agents. We would not be suspected of an intention to criticise, in any particular, the method of Berzelius and the results it has produced, especially in what pertains to the platinoid metals. Since we have obtained a complete knowledge of his researches in this direction, we confess that he has left very little to do in the way he had marked out. Those who choose to give themselves the trouble to read with attention all that concerns this question in the *Traité de Chimie*, by Berzelius, will see that he left to M. Claus the distinguished honor of discovering ruthenium only because he operated on insufficient quantities of material.* After Berzelius and Wöhler, after MM. Claus, Fremy, Fritzsche, and other skillful chemists who have studied platina and the platinoid metals by the ordinary processes of the humid method, there remained to us, in order to obtain new facts, no other resource than an entire change of method; this is what we have done, and we proceed to give the results we have obtained.

### I.—Osmium.

Osmium has been hitherto prepared in a form which leaves the account of its physical properties as imperfect as if iron had been only known as pyrophoric iron, or silicium and borax in no other condition than that of amorphous and highly combustible substances. We have considered osmium as a metalloid; and indeed, like some other metalloids, it changes entirely its chemical and physical properties in accordance with the manner in which it has been prepared. Common osmium, prepared by the processes of

* Berzelius (see the first French edition of 1831, vol. iv, pages 456 and 457,) gives the mode of preparing ruthenium by one of the processes recommended by M. Claus; he finds the rose-colored salt, $Ru_2\ Cl_3$, 2Kcl, characteristic of ruthenium, and the composition of the chloride $Ru_2\ Cl_2$: mentions its analogy with and its difference from the chloride of rhodium, with which he compares it, concludes that it is not rhodium, on account of its resistance to bisulphate of potash, and only admits that it is a sub-iridic chloride, because, "when the metal extracted from it by hydrogen is warmed in chlorine gas with chloride of potassium, it gives only the common iridico-potassic chloride, so that it is impossible to attribute its existence to the presence of a foreign metal mingled with iridium. Besides osmium can form similar rose-colored salts."

Berzelius, is a spongy semi-metallic mass, exhaling a very sensible odor of osmic acid, which indicates an appreciable alteration by oxygen at common temperatures. Its density is equal to 7. If obtained by reducing a mixture of hydrogen with the vapor of osmic acid, as Berzelius did, it is metallic, and has a density of about 10.

*Pulverulent Osmium.*—Osmium appears with totally different characters if we prepare it in the following mode. Fine osmide of iridium, sifted through gauze, is used; if that which is naturally pulverulent cannot be obtained, it is chemically divided by a process hereafter to be described in the chapter devoted to ruthenium. One part of divided osmide is mixed with five times its weight of binoxyd of barium, which has been weighed with great care, so as to be able afterwards completely to precipitate it with a known weight of sulphuric acid. This mixture rendered as intimate as possible by prolonged rubbing in a porcelain mortar, is warmed for one or two hours at the fusing point of silver, in an earthen crucible, which has been closed as perfectly as possible by a cover carefully fitted and luted with a little fire clay. After the experiment we find a black homogeneous substance, which is roughly divided and introduced into a glass retort, the beak of which should be ground if possible. A little water is first poured on, then eight parts of muriatic and one of common nitric acid; after which it is distilled, special attention being paid to cooling the receiver, which should be fitted to the retort with the greatest care, to avoid loss of osmic vapor. The operation is finished when the vapor taken at the tubule of the retort no longer possesses the characteristic odor of osmic acid. The liquid contained in the recipient is then distilled a second time, and the product may then be collected in dilute ammonia introduced in the tubulated balloon into which passes the product of the second distillation. The osmiate of ammonia is supersaturated by sulphuretted hydrogen, and the liquid containing the sulphuret boiled a long time and then filtered. The filter must not be dried at too high a temperature, or the sulphuret of osmium will take fire and the substance almost entirely disappear, being converted into osmic and sulphurous acids. The sulphuret is introduced into a crucible of gas-coke, very smooth on the inside, and furnished with a cover which is rubbed to fit closely. This is then placed in a crucible of refractory clay. Between these two crucibles sand is poured, the clay crucible is well covered, and the whole kept for four or five hours at the fusion point of nickel.* The sulphuret of osmium is reduced by the heat and leaves a brilliant metal of a clearer blue color than zinc, in small fragments which are very easily separated. If it is desired still more metallic and more dense, it must be heated at the melting point of rhodium, in an apparatus which we shall presently describe. Then its density is 21.3. Sometimes it is obtained at 21.4; that is to say, it is equal and even superior in density to platina.

This osmium is inodorous and can be heated to the melting point of zinc without giving off vapors of osmic acid. At a higher temperature, however, it becomes combustible.

*Crystallized Osmium.*—When osmium is dissolved in tin by heating it to bright redness with seven or eight times its weight of tin, in a charcoal crucible; if the metallic mass be allowed to cool slowly, osmium separates at the moment of chilling, as boron and silicium separate from aluminum or from zinc, that is, by crystallizing. The tin may then be dissolved out with muriatic acid, leaving a very hard crystalline powder which retains no tin, while the acid does not easily dissolve osmium.

*At the Normal School we used to produce these high temperatures with fragments of gas coke too hard to be cut for elements of galvanic batteries. This coke, which leaves no cinder, softens those crucibles which are of bad quality, but does not destroy their walls like the clinker of common coke. It kindles with difficulty, but burns with extraordinary energy.

| We have taken | I. | II. |
|---|---|---|
| Osmium, . . | 4.00 grs. | 4.50 grs. |
| And mixed it with | | |
| Tin, . . | 24.00 grs. | 24.00 grs. |
| After dissolving we have recovered | | |
| Osmium crystallized, | 3.96 grs. | 4.60 grs. |

The crystals of osmium are too small to be measured. Upon some, under the microscope, are seen two kinds of very distinct faces, rhombs and squares, so arranged in relation to each other, as to lead to the supposition that the crystals are rhombic dodecahedra with the faces of the cube.

*Compact Osmium.*—An alloy of the same kind may be made with zinc, but the osmium separates in an amorphous condition, or rather the alloy is not broken up by the chilling of the metal; for if the zinc is dissolved out by muriatic acid, there remains an amorphous powder of great combustibility, which is pure osmium, and the acid does not take up a sensible quantity of osmium.

| | |
|---|---|
| We have taken | |
| Osmium, . . . | 8.50 grs. |
| And mixed it with | |
| Zinc, . . . . | 50.00 grs. |
| After solution we have recovered | |
| Osmium, . . . | 8.60 grs. |

But if, instead of dissolving this alloy, we drive off the zinc by a very high temperature and finally subject it, in a charcoal crucible, to the heat developed in a furnace of explosive gas, (such as we shall presently describe,) capable of liquefying rhodium, we shall find the osmium perfectly metallic, with a lustre and bluish tint characteristic of this metal. This metal, very compact though it undoubtedly is, has not yet been melted: for it is full of irregular cavities which will be rounded even by the simple effect of softening. These cavities diminish the density of the matter, because they do not fill with water, not being always in communication with the exterior.

This osmium is then very hard, easily scratching glass.

The matter contains no zinc, even when it has been heated only to the temperature of melting cast iron.

We have taken

| | |
|---|---|
| Osmium, . . . . | 4.35 grs. |
| Zinc, . . . . | 25 00 grs. |

And have recovered after volatilizing the zinc

| | |
|---|---|
| Osmium, . . . . | 4.30 grs. |

Osmium has so little tendency to combine with metals, that on heating tin with osmide of iridium, and attacking the alloy with muriatic acid, we get crystallized osmium in fine powder, and a compound of iridium and tin, crystallized in cubes, which will hereafter be considered.

We have attempted to melt osmium at a temperature which we suppose corresponds to the fusing point of rhodium, we have availed ourselves of the following apparatus.

It is composed of a blowpipe, E E′, C C′, fig. 1, of a furnace, A B D, and of a crucible, G H J, in which the osmium is put.

The blowpipe is composed of a copper cylinder, E E, 12 millimetres in diameter, terminated below by a slightly conical platina jet, 40 millimetres long. A copper tube C C C, 3 or 4 millimetres in internal diameter, and terminated by a platina button, C′, which is adjusted by a screw, enters the first cylinder from above, and is kept in place by a screw-cap, P, which enables us, by loosening it, to adjust the button C′, to any desirable height in reference to the inferior extremity of the cylinder, E E, E′ E′.

A cock, H, is applied laterally with a very large tube to the cylinder, E. A cock, O, terminates the bent extremity of the tube, C. By the stopcock, H, hydrogen or illuminating gas is brought to the apparatus, and oxygen is admitted by the stopcock O. The button, C′, is pierced by a hole varying in diameter from 2 to 3 millimetres according to the dimensions of the apparatus to be constructed. We generally use one of 2½ millimetres.

FIG. 1.

The furnace, A B D, is composed of three pieces of well burned quicklime, slightly hydraulic, and just compact enough to admit of being turned. There is nothing gained by using very hard lime which the tool does not readily cut. The lime we use is very common in Paris, and comes from the calcination of the *calcaire grossier* of the tertiary deposits of Paris. A first cylinder, A A, is pierced by a slightly conical opening in which the blowpipe fits tightly to half its thickness, the button, CC′, reaching to within 2 or 3 centimetres of the lower opening of this hole. A second cylinder of lime, B B, is pierced by a cylindrical hole much larger than the first, the dimensions of which are such that it should leave between its walls and the crucible, H H, a distance of 3 or 4 millimetres at most. Its height is a little greater than that of the crucible. A third cylinder, D, on which the second rests, is grooved upon its upper surface by four deep furrows, K K, at right angles to one another, which give exit to the products of combustion. At the centre of this upper surface, and firmly attached to the substance of the cylinder, is placed a little support, D′, on which rests the crucible.

The crucible itself is made as follows: a cylindrical piece of lime, H H, hollowed out to receive a smaller crucible, I, made of gas coke, and furnished with a cover in which is placed the matter to be heated.

The lime crucible is surmounted by a conical top, G, the summit of which ought to be directly under the button of platina, C′, at a distance of two or three millimetres, varying with the rapidity of the current of gas. This cone, G, is so made to force the flame of the blowpipe to spread itself equally around the crucible, H, then to issue through the openings, K.

All the cylindrical pieces, A, B, D, ought to be strongly bound with very soft iron bands placed at short distances apart, to sustain the lime which always splits a little during the heating.

To use the apparatus, (the osmium having been introduced into the coke crucible,) the crucibles are adjusted on the base, D; then the piece A is raised with the blowpipe, of which the stopcock, H, for the passage of the combustible gas, is open. The gas in C is kindled, and oxygen gradually admitted by opening the stop cock, O, so as to allow the combustible gas to predominate considerably; then, introducing the flame into the apparatus, all is arranged as indicated in the figure. By means of the screw cap, P, C′, is properly adjusted and kept in position for an indefinite time by tightening the screw. The speed of the oxygen and hydrogen currents is then gradually augmented till the maximum temperature is attained. This is directly determined by looking through the apertures of the apparatus, and by attending to the noise made by the blowpipe. This noise should be as weak as possible when the volumes of the gases are in proper proportion. When everything is well regulated, at the end of eight minutes the crucible, to its very centre, reaches the melting point of rhodium.*

* At these temperatures, the contact of lime and carbon cannot be indefinitely prolonged without the mutual destruction of these bodies, by the formation of

Osmium treated in this apparatus has a very well marked metallic lustre, with a bluish tint, less decided than when it has been slightly heated. Its density is 21.4, and its properties have already been described. But it presents no trace of fusion. On the other hand, during the entire progress of the experiment, no odor of osmium is perceived in the flame, provided the crucible remains intact. We can then say, that at the temperature of melting rhodium, and in close vessels, osmium is infusible and fixed.

*Volatility of Osmium.*—The case is not the same at a higher temperature, that, for example, at which ruthenium enters into full fusion; which can only be obtained by means of the blowpipe with mixed gases, that we shall presently describe. When osmium is submitted to the action of this flame, which to have its full effect, should be neither oxydating nor reducing, we see, at the precise moment when the heat attains its maximum, considerable quantities of osmium disappear with great rapidity, and deposit themselves as soot upon any object placed in the immediate vicinity of the flame. Osmium can burn then, and does burn, but the operator can readily detect its volatilization. It is a very interesting experiment which should be repeated but with extreme precaution. One of us who has made it twice, was blind for nearly twenty-four hours, in consequence of the current of air, which should have protected him against the copious vapors of osmic acid, having changed its direction and unexpectedly driven these fumes oxyd of carbon and of calcium, the presence of which becomes manifest in the flame. At the point of contact of the carbon and lime, the latter is deoxydated, gives out the odor of hydrogen, and burns even in water when introduced into that fluid. We can but indicate these phenomena among those which we study at this time with the apparatus we have just described, and with tubes constructed in an analogous manner, in which we are studying a great number of chemical reactions at the melting point of rhodium. It suffices to announce that we hope in such conditions to realize a great number of reductions hitherto considered impossible, especially the reduction of baryta by carbon, boron, &c. We desire here only to claim priority for these experiments and to give ourselves the opportunity of continuing them long enough for the complete development of all the facts.

upon him. He then felt in his eyes a pain equivalent to that of a severe blow. The osmium, reduced on the surface of the conjunctiva, does not subsequently disappear, and so contributes to render the sight indistinct. We conclude from these experiments, that at the melting point of iridium, when platina itself vaporizes, osmium becomes volatile. But even then the practiced eye which follows attentively the phenomenon cannot detect in the osmium which rapidly disappears, the slightest trace of fusion. Osmium must be heated in vacuo, by the aid of a powerful voltaic current, before the problem can be definitely solved.

Fig. 2.

Experience has taught us that the maximum temperature which can be obtained with explosive gases, is obtained by mixing them in advance and burning them at the extremity of a blowpipe, at a distance of 3 or 4 millimetres at most from this extremity. To determine without danger to the operator, these favorable circumstances, we have contrived the following little instrument.

The gases, produced directly or contained in gasometers, are separately introduced into an apparatus, the interior volume of which is so small that the report of the explosion is scarcely perceptible, and all danger is avoided.* The hydrogen gas arrives by the stopcock H, and passing through the socket D, expands in a tube of caoutchouc in the interval, H′, whence

*One of us brought back from London, in 1856, a beautiful instrument made by Mr. Ansell, which gave us the idea of our apparatus The blowpipe of Mr. Ansell, more complicated, less portable, but more elegant, is admirably suited to demonstrations in an amphitheatre.

it arrives by a second socket, E, in a bent tube, O′ E′ B, where it is mixed with oxygen. As to the latter, it enters by the stopcock O, passes by the socket E, in a special copper tube which opens in O″, where the gas is mixed with hydrogen. The explosive gas is lighted in B at the extremity of a large button of copper, strong enough sufficiently to chill the gas. With a little skill, this blowpipe can be held by the socket, D, with the same hand which regulates at will the two stopcocks, O and H.*

MM. Thenard and Dulong had already found that osmium possesses the same property which they established for platina, of determining the combination of substances by mere contact with the reacting matter. We have established the existence of this property not only in osmium, but also in the other metals of platina, in which it exists to a very marked degree.

The spontaneous decomposition of sulphide of osmium by heat can be demonstrated by the experiment we have related, in which we prepared osmium by calcining the sulphide in a charcoal vessel. But as it might be supposed that the affinity of carbon for sulphur determined this reduction, we put the sulphuret in an earthen crucible enclosed in one of charcoal, which exhales no hydrogen gas, as may be the case in brasque of wood charcoal, and which hinders the roasting of the sulphide. In such conditions the decomposition of the sulphide of osmium is readily accomplished. This observation is equally applicable to gold as well as to the other platinoid metals, and we shall find that it has great interest for us, because we base upon this curious property one of the methods of separation employed by us for the analysis of the ore and residue of platina.

*Osmic Acid.*—Dry osmic acid is easily prepared by the roasting process indicated by M. Fremy, which succeeds

* The maximum temperature is reached when the two gases burn without making the least noise. With an excess of hydrogen the flame *blows*, (*souffle;*) with an excess of oxygen it *whistles*, (*sifle.*)

sometimes well, sometimes ill, according to the nature of the osmides. When these roast easily, this method must be employed as its author has described it. If not, the osmides are rendered easy of oxydation by destroying their aggregation in the following manner: The osmides are mixed with eight or ten times their weight in zinc, and the whole digested for several hours at bare redness; when the solution of the osmium in the zinc is complete, the alloy is treated with muriatic acid, which leaves a powder so combustible that it gives off osmic acid at the common temperature, and takes fire at about 400°, giving off osmic acid and oxyd of zinc. It is even necessary, before introducing this powder into the apparatus of M. Fremy, to calcine it to dull redness to diminish its combustibility. This powder is a mixture of finely divided osmium and an alloy of iridium and zinc, in which, consequently, the primitive elements of osmium are entirely separated. The following results were obtained with osmides rich in osmium.

Osmide in scales treated with zinc, gave a compound of

| | | |
|---|---|---|
| Osmium and iridium, | 82.6 | |
| Zinc, alloyed with iridium, | 17.4 | |
| | 100.0 | |
| Loss of osmium by wasting, | 30 | per cent. |
| Corresponding osmic acid, | 39.7 | " |

Russian osmides in grains, which yielded no osmic acid by roasting, can furnish 18 or 20 per cent. of it after being disaggregated by zinc. We find, moreover, on applying the most delicate tests for osmium, that osmides thus treated retain but insignificant traces of this metal.

*Density of the Vapor of Osmic Acid.*—The formula of osmic acid, $Os\,O_4$, which really plays the part of an acid, has a sort of exceptional composition among the acids now known to us; so that it was of some importance to determine the density of its vapor, which is moreover, an easy task, since osmic acid boils at about 100°, is readily manipulated, and can be obtained in a state of perfect purity.

We have prepared osmic acid by the process indicated by Berzelius, that is, by roasting pure osmium in oxygen. It was introduced into a balloon with a long neck, full of dry and weighed air ; the neck is then drawn out, and the useless parts cut away, returned to the balance. The balloon, introduced into an oil bath, was raised to 245.2°, (246° corrected,) and the following results were obtained :

| | | |
|---|---|---|
| Temperature of the balance, | 19° | |
| Pressure during the operation, | 764.55 | millimetres. |
| Temperature of the barometer, | 16.° | |
| Temperature of the oil bath, | 245.2° | |
| Excess of weight, . . | 1453 | milligrammes. |
| Residual air, . . | 0 | |
| Volume of balloon, . . | 299 | centimetres. |
| Weight of the litre, . . | 11.48 | grammes. |
| Density, . . . . . | 8.89 | |

The temperature of 246° is sufficiently above the boiling point of osmic acid to allow us to rely on this experiment. Nevertheless, we concluded to recommence the operation at a temperature notably higher, to determine that this acid is not in the category of acetic acid, and to guard against the cause of error pointed out by M. Cahours*

The following are the new elements :

| | | |
|---|---|---|
| Temperature of the balance, . | 19.5° | |
| Barometer at the moment of the tare, . . . . . | 762.5 | millimetres. |
| Temperature of the barometer, | 17.5° | |

* The peculiarity alluded to is that the density of acetic acid vapor, determined at 152°, is 2.78, corresponding to 3 volumes ; whereas, when determined at 230°, it is 2.09, corresponding to 4 volumes.—*Translator.*

| | | |
|---|---|---|
| Barometer at the moment of closing the balloon, . . . | 762.2 | millimetres. |
| Temperature of the barometer, | 17.5° | |
| " of the oil bath, . | 285.0° | |
| " " corrected, | 286.0° | |
| Excess of weight, . . . . | 977 | milligrammes. |
| Residual air, . . . . | 1 | cubic centimetre. |
| Volume of balloon, . . . | 220 | " " |
| Weight of litre, . . . | 1147 | milligrammes. |
| Density of vapor, . . . . | 8.88 | |

that is identical with the first determination.

The density calculated by the formula,

$$131.5 \times 0.0692$$

gives the number 9, which is, it is true, very near 8.9. These numbers prove that osmic acid, $OsO_4 = 131.5$, represents two volumes of vapors, a fact which is remarked in a great number of volatile matters in mineral chemistry.

Although the numbers 8.859 approximate closely, they differ in an unusual way, since the number determined by experiment is commonly larger than the theoretic amount: we must therefore admit either that there is a disturbing cause peculiar to osmic acid, upon which this difference depends, and which we may have neglected, or else that the equivalent of osmium should be slightly lowered. There is no other apparent cause of error in this operation; no reduction of osmium, every thing remaining in the balloon being either crystallized or gaseous. A remarkable peculiarity is observed at the moment the balloon is opened under mercury. By contact with osmic acid, the mercury acquires the property of moistening the glass, and the balloon is perfectly coated with reduced osmium, or rather with an amalgam of that metal.

(Continued in our next.)

ERRATA.—The translator not having seen the proof of the first part of this article, finds that some errors have been made in the proper names, which the reader will please correct. Page 195, 10th line from bottom, for Kocksbarrow read *Kocksharrow;* next line, for Rochette read *Rachette;* 2d line from bottom, for Desmontes read *Desmoutes;* bottom line, for Pavard read *Savard.*

## Figures and Tables

**Fig. 1. f1:**
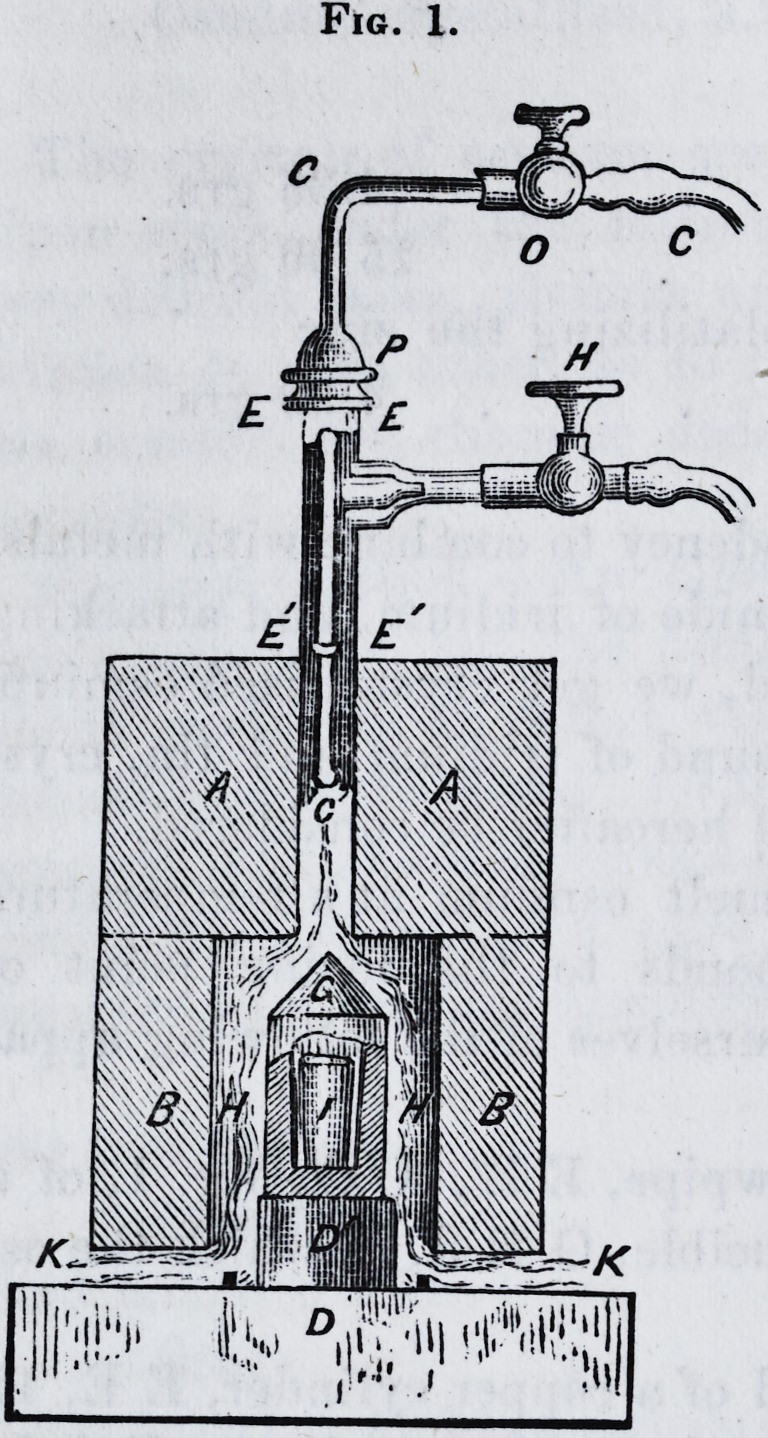


**Fig. 2. f2:**